# Co-crystallization in Antiepileptic Drugs: A Path Toward Better Therapeutic Outcomes

**DOI:** 10.7759/cureus.82230

**Published:** 2025-04-14

**Authors:** Bhagyashree Patil, Sanjay Surana, Atul Shirkhedkar

**Affiliations:** 1 Pharmaceutics, R. C. Patel Institute of Pharmaceutical Education and Research, Shirpur, IND; 2 Pharmacognosy, R. C. Patel Institute of Pharmaceutical Education and Research, Shirpur, IND; 3 Pharmaceutical Chemistry, R. C. Patel Institute of Pharmaceutical Education and Research, Shirpur, IND

**Keywords:** advanced methods, antiepileptic drug, crystal engineering, future scope, pharmaceutical cocrystals

## Abstract

Pharmaceutical cocrystals are highly valued in drug development as they can enhance active pharmaceutical ingredients (APIs)' physicochemical characteristics without changing their chemical structure. This is especially important for biopharmaceutical classification system (BCS) class II and IV drugs, which have poor water solubility, resulting in low bioavailability. Co-crystallization is the process of forming a crystalline solid where the API and a co-former are combined in a specified stoichiometric proportion within a crystal lattice, mainly stabilized by non-covalent interactions. This method can enhance properties like stability, dissolution rate, solubility, compressibility, and powder flowability and pharmacokinetics, resulting in an improved drug delivery system and therapeutic effectiveness. Pharmaceutical antiepileptic drugs (AEDs) are the main focus of this review. Pharmaceutical characteristics, conventional and advanced cocrystal generation, and assessment techniques, as well as recent developments related to cocrystals, may suggest perception for cocrystal potential, design approaches, and regulatory aspects. The study’s overall finding emphasizes the growth of co-crystallization as a significant technique to enhance the drug’s performance and also demonstrates its potential as a significant technique in the AED category and its future application.

## Introduction and background

Pharmaceutical products are vital commercial commodities in our daily lives, playing a crucial role in maintaining health and treating diseases. About 80% of the drugs used today are utilized in solid forms, as most active pharmaceutical ingredients (APIs) are crystalline solids at room temperature. Moreover, oral ingestion is the most expedient and commonly used route for drug delivery because of its ease of administration, high patient compliance, least sterility constraints, cost effectiveness, and flexibility in dosage form design [1]. Even though enormous efforts and capital are spent on discovering and developing new drugs, the successful candidates often show poor physicochemical properties [2]. Solubility and permeability are fundamental properties that affect bioavailability and dosage form. Because of the new chemical entities that are insoluble in water, there has been an increasing interest in designing strategies that can enhance the solubility of drug molecules without changing their molecular structure activity [3].

**Figure 1:**
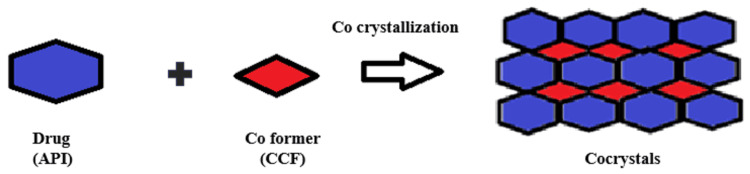
Cocrystal schematic diagram The image was created by the authors using Microsoft Paint (Microsoft Corp., USA).

Co-crystallization is an effective strategy for altering the physicochemical characteristics of APIs to enhance stability, solubility, dissolution rate, and bioavailability [4-8]. Many newly discovered drugs show low aqueous solubility, which presents a significant challenge in the development of oral dosage forms [6,8]. Co-crystallization entails the combination of an API with a co-former, illustrated in Figure 1, in the stoichiometric proportion within a crystal lattice, stabilized by non-covalent interactions [8,9].

This method provides a valuable opportunity to enhance the physicochemical properties of drugs without altering their intrinsic pharmacological activity. Pharmaceutical cocrystals offer an alternative strategy for expanding the variety of solid pharmaceutical ingredients beyond traditional methods like polymorphs, salts, and solvates [9]

Researchers are exploring a number of strategies for synthesizing cocrystals, including solid-state techniques like grinding and solution-based methods such as slow evaporation [10]. Choosing appropriate co-formers depends on considerations like hydrogen bonding, molecular recognition features, and their solubility in water. Cocrystals provide multiple benefits, such as increased solubility and dissolution rates; greater stability, especially for APIs sensitive to moisture; and altered mechanical properties that enhance tablet ability [11]. Cocrystal research is an ever-evolving field, with progress being made in areas like polymorphic cocrystals, higher-order cocrystals, and the thermodynamics of co-crystallization. Regulatory bodies in the United States and Europe have provided guidelines on pharmaceutical cocrystals, facilitating the registration and development of drug products based on cocrystals [12].

Several researchers have dedicated their efforts to designing and preparing pharmaceutical cocrystals that can improve the solubility of biopharmaceutical classification system (BCS) class II and IV drugs [8,13]. Although the cocrystal field has been well studied worldwide throughout the last decade of the 20th century, there are limited published works available on antiepileptic drugs (AEDs). Therefore, this paper aims to determine the extent to which the crystallization approach has been used to improve the solubility of some of these poorly soluble AEDs. This paper first briefly discusses a review of some pharmaceutical cocrystals of carbamazepine, gabapentin, pregabalin, lamotrigine, valproic acid, and oxo-carbamazepine, followed by the influence of co-crystallization on drug properties, synthesis, characterization methods of cocrystals, and future trends in cocrystal research.

Impact of co-crystallization on drug properties and performance

Co-crystallization has evolved into a successful technique to modify the physicochemical properties of drugs, offering significant improvements in their performance without altering their inherent pharmacological activity [14]. Hence, its utility in overcoming challenges associated with drug solubility, dissolution rate, stability, and manufacturability, ultimately leading to enhanced drug delivery and therapeutic efficacy [15,16].

Co-crystallization has become a valuable method for altering the physicochemical properties of drugs, providing notable enhancements in their performance while preserving their fundamental pharmacological activity [17,18]. The literature highlights its effectiveness in addressing issues related to drug solubility, dissolution rates, stability, and manufacturability, ultimately resulting in improved drug delivery and therapeutic effectiveness [19,20].

## Review

AEDs decrease the seizure frequency and severity in patients with seizure disorder, epilepsy, and epilepsy syndrome. These AEDs can be divided into older medications, i.e., first generation, and newer medications, i.e., second and third generations. All AEDs except fosphenytoin are available in oral formulations with various dosing frequencies; hence, for drugs with limited solubility and /or permeability issues, there is a scope to improve their bioavailability and antiepileptic efficacy through co-crystallization approaches [21-24].

Reported cocrystals of AEDs

**Figure 2:**
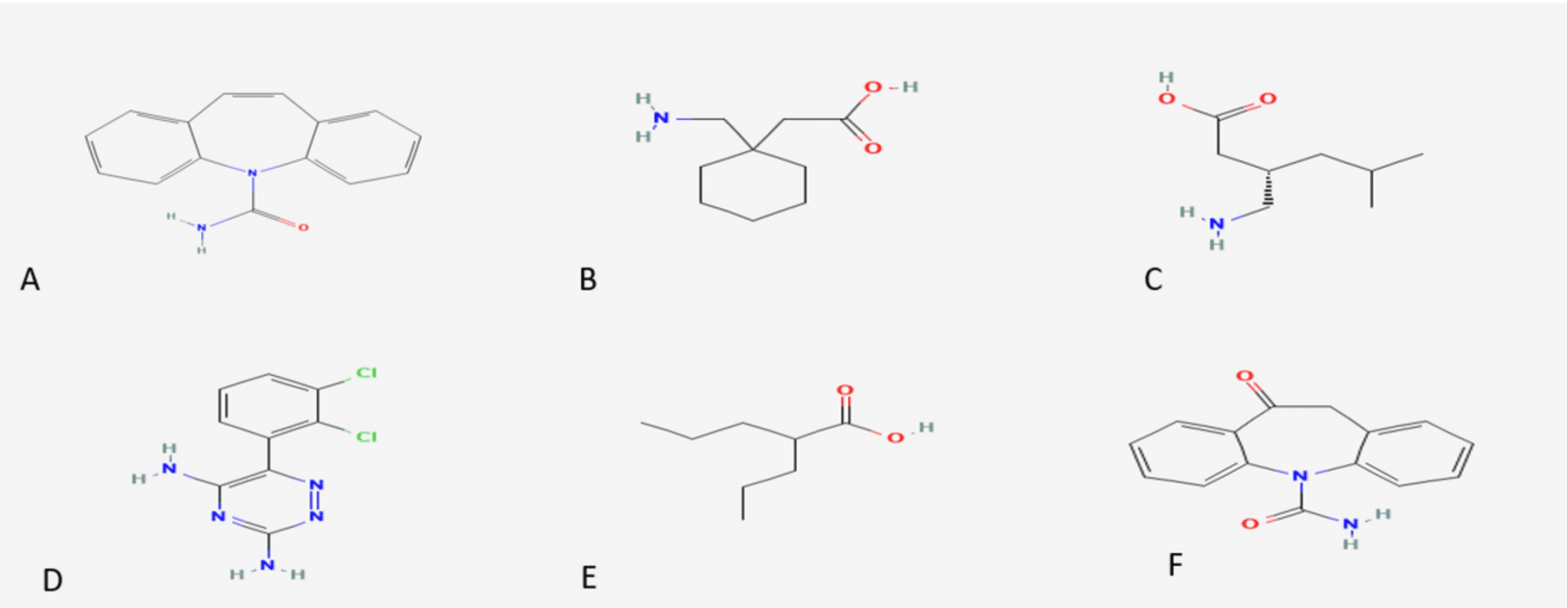
Structures of antiepileptic drugs (A) National Center for Biotechnology Information (2025). PubChem compound summary for CID 2554, Carbamazepine. Retrieved March 12, 2025, from https://pubchem.ncbi.nlm.nih.gov/compound/Carbamazepine. (B) National Center for Biotechnology Information (2025). PubChem compound summary for CID 3446, Gabapentin. Retrieved March 12, 2025, from Gabapentin | C9H17NO2 | CID 3446 - PubChem. (C) National Center for Biotechnology Information (2025). PubChem compound summary for CID, 5486971 Pregabalin. Retrieved March 12, 2025, from Pregabalin | C8H17NO2 | CID 5486971 - PubChem. (D) National Center for Biotechnology Information (2025). PubChem compound summary for CID, 3878 Lamotrigine. Retrieved March 12, 2025, from Lamotrigine | C9H7Cl2N5 | CID 3878 - PubChem. (E) National Center for Biotechnology Information (2025). PubChem compound summary for CID, 3121 Valproic acid. Retrieved March 12, 2025, from Valproic Acid | C8H16O2 | CID 3121 - PubChem. (F) National Center for Biotechnology Information (2025). PubChem compound summary for CID, 34312 Ox-carbamazepine. Retrieved March 12, 2025, from Ox-carbamazepine | C15H12N2O2 | CID 34312 - PubChem.

In recent years, multiple cocrystals have been developed using AEDs, with some receiving FDA approval and becoming commercially available. As per the latest Food and Drug Administration (FDA, 2018) and European Medicines Agency (EMA, 2015) guidelines, the approval process for cocrystals has been streamlined, making regulatory approval more accessible. The application of crystal engineering techniques to enhance drug solubility has gained significant attention, with co-former solubility emerging as a widely adopted strategy. Co-former solubility is often used as a predictive factor for the solubility enhancement of cocrystals. Studies have reported varying degrees of correlation, including complete, partial, or even negative correlations. In addition, research suggests that cocrystals are more likely to form when the ΔpKa falls within the range of -1 to 4. This review focuses on the advancements in improving the solubility of AEDs (Figure 2) while also addressing enhancements in their physicochemical properties [25].

Carbamazepine

Carbamazepine is an AED having low bioavailability due to its hydrophobic nature, measuring below 70%, along with inconsistent oral absorption and limited water solubility. Moreover, carbamazepine has a narrow therapeutic window, and its bioavailability varies. Over the past decade, various studies have been conducted on carbamazepine (CBZ), a BCS class II drug, to enhance its solubility. The limited bioavailability of CBZ is primarily attributed to its poor solubility. Its chemical structure is depicted in Figure 2A. Since the drug's absorption is dissolution-limited, resulting in low oral bioavailability, achieving the desired therapeutic effect often requires administering CBZ in high doses. There have been reports of various cocrystals with dicarboxylic acid co-formers. Based on the reported findings, CBZ-GA exhibited significant enhancement in dissolution, making it the most soluble yet unstable cocrystal [26].

Mohammad et al. reported cocrystals prepared by the slurry co-crystallization technique with adipic acid, glutaric acid, succinic acid, and malonic acid [27]. The study shows the influence of the spacer group (varying aliphatic chain) on solubility, dissolution, RH stability, and oral bioavailability of CBZ cocrystals with a dicarboxylic acid conformer. Similarly, Shayna et al. reported that CBZ-cinnamic acid cocrystals demonstrated enhanced solubility and dissolution rates compared to pure carbamazepine (CBZ) [28].

Recently, several drug-drug cocrystals (DDCs) involving carbamazepine have been reported. Majumder et al. reported a 1:1 DDC of carbamazepine and indomethacin [29]. Nicolai et al. reported carbamazepine and aspirin [30]. Drozd et al. reported carbamazepine with 4-aminosalicylic acid [31]. Numerous patents have been granted or filed pertaining to the development and characterization of carbamazepine cocrystals, reflecting their pharmaceutical relevance and industrial interest [32,33].

Gabapentin

Gabapentin (GBP) (1-(aminomethyl) cyclohexane acetic acid) is an antiepileptic medication that structurally resembles the neurotransmitter γ-aminobutyric acid (GABA). Its chemical structure is depicted in Figure 2B. Initially developed under the brand name Neurontin for epilepsy treatment, gabapentin is now extensively used for managing pain, particularly neuropathic pain. It is generally well tolerated by most patients and has a mild side effect profile. Gabapentin is a water-soluble, bitter-tasting, white crystalline compound with a saturable absorption mechanism. It has been reported to exhibit high water solubility, low permeability, and no protein binding and is not metabolized by the liver [34]. However, due to its short half-life (four to six hours) and limited absorption, the development of new formulations is necessary to address these challenges.

Samineni's work reports the formulation and evaluation of gabapentin cocrystals with various co-formers, like B.A, S.A, and tartaric acid by solvent drop, co-grinding, and solvent evaporation method. In this work, solvent evaporation produces the best results compared to other methods. The prepared multicomponent co-crystal formulation and pure gabapentin were evaluated for saturation solubility analysis; pure gabapentin showed 5.99 mg/ml and GBP-TA cocrystals showed a high solubility value of 8.10 mg/ml and 13-fold solubility increases compared to pure drug [35].

Reddy et al. predicted pH-dependent cocrystal solubility and stability of gabapentin [36]. Soliman et al. reported that co-crystallization with saccharin can modify the physicochemical properties of a drug and influence its in vivo absorption [37].

*Pregabalin * 

In Kamisarek et al.'s study, different co-crystalline forms of pregabalin enantiomers with mandelic acid as co-formers were investigated [38]. Pregabalin demonstrates a unique ability to form either a zwitterionic or neutral co-crystal with mandelic acid, as initially reported by Samas et al. in 2007 [39]. In addition, the nature of the resulting system - whether composed entirely of charged molecules or not - depends on whether homo- or heterochiral co-formers are used in the co-crystallization process hence in 1:3 systems, no proton transfer occurs between co-formers when a homochiral configuration (S, S) or (R, R) is present, depicted in Figure 5. However, in species with (S, R) or (R, S) chirality, each molecule becomes formally charged in pregabalin:mandelic acid co-crystalline structures. Homo- and hetero-pregabalin-mandelic acid species exhibit a remarkably different solubility and melting behavior based on molecular charge differences, even though they are structurally very similar. Pregabalin's chemical structure is depicted in Figure 2C [38].

Lamotrigine

Lamotrigine is an AED from the phenyl triazine class and is categorized as a BCS class II drug. Lamotrigine faces a significant challenge due to its low aqueous solubility (0.17 mg/mL at 25°C), resulting in poor solubility within gastrointestinal fluids. Co-crystallization presents a promising approach to enhance its physicochemical properties and improve its performance [40]. It is commonly prescribed for the treatment of epilepsy and bipolar disorder and also functions as a mood stabilizer. Its mechanism of action involves selectively binding to inactive sodium channels, thereby inhibiting sodium currents and reducing the release of the excitatory neurotransmitter glutamate. Its chemical structure is depicted in Figure 2D [41].

The objective of Samineni et al. was to formulate lamotrigine cocrystals. 4-hydroxy BA, saccharin sodium, and methyl paraben were used as cocrystal formers to form cocrystals of lamotrigine. These cocrystals were used to treat simple and complicated partial seizures and generalized tonic-clonic seizures that are resistant to multiple medication treatments, and the selected formulation of cocrystals shows good retention characteristics, which will ultimately improve the clinical response [42].

Valproic Acid

For some drugs, cocrystal forms were developed at a later stage, and for some others, they were identified as a cocrystal after some years of approval. Valproic acid is an approved medication for epilepsy [43]. Valproic acid is naturally a liquid, and its solidification is essential for developing solid dosage forms. The most common solid form is its sodium salt, sodium valproate. While sodium valproate has been widely utilized in various formulations, it presents a challenge due to its physical instability. Its chemical structure is depicted in Figure 2E. It is highly hygroscopic and readily absorbs moisture, even under standard room conditions. As per Khajir et al., liquid valproic acid is solidified using tromethamine, which exhibits lower hygroscopicity compared to sodium valproate. The cocrystal form of (1:1) ratio is less hygroscopic than the pure component. Depakote is a pharmaceutical product that serves as evidence of a commercially available drug containing a cocrystal-based active pharmaceutical ingredient (API).

Oxcarbamazepine

Oxcarbazepine (OXCBZ, marketed as Trileptal) is a contemporary AED used both as monotherapy and adjunctive therapy for treating partial seizures. This neutral, lipophilic compound has a melting point of 215-216°C and a molecular weight of 252.268 g/mol. Despite its therapeutic potential, oxcarbazepine exhibits very low bioavailability due to its poor solubility. To enhance its dissolution rate, various formulation strategies have been explored, including complexation with hydroxypropyl β-cyclodextrin, microcrystal formation with methylcellulose, granulation with solubility and release-enhancing agents, and solid dispersions. Co-crystallization is an effective approach for enhancing physicochemical properties, including solubility and dissolution, thereby increasing the potential of non-salt-forming APIs [44]. It has an amide functional group and carbonyl group in its structure, its chemical structure depicted in Figure 2F, which used to form cocrystals by liquid assisted grinding using oxalic acid,2,5 dihydroxybenzoic acid and salicylic acid. The apparent solubility of OXCBZ-OA and OXCBZ-2,5-DHBA cocrystals increased approximately 2.6 and 4.7 times than that of the pure drug [45]. Similarly, Chadha et al. reported that compared to pure OXCBZ, the cocrystal with saccharin exhibited a lower ED50 value and a significant enhancement in OXCBZ's solubility under aqueous conditions [46].

Co-crystallization influences key drug properties

Solubility and Dissolution Rate

A key reason for co-crystallization is to enhance the solubility and dissolution rates of drugs [47], especially those categorized as BCS class II and IV. This is accomplished by choosing co-formers that increase the solubility of the resulting cocrystal compared to the pure drug. The solubility of the co-former is often related to the dissolution rate of the cocrystal. However, the literature also warns that co-crystallization does not always lead to improved solubility. The selection of a co-former and the resulting crystal packing play an important role in influencing the solubility characteristics of the cocrystal [48]. In certain instances, cocrystals may demonstrate lower solubility compared to the parent drug. However, this could seem counterintuitive, it can be beneficial for applications involving controlled or sustained drug release.

Stability

Co-crystallization provides an effective strategy for tackling both physical-chemical stability concerns related to drugs [19]. Co-crystallization can help alleviate physical stability issues, especially the tendency of certain drugs to develop hydrates when exposed to humidity. For instance, caffeine, which is susceptible to hydrate formation, can be stabilized against moisture “by forming cocrystals with oxalic acid or malonic acid” [49]. Co-crystallization can also impact chemical stability. The varying spatial arrangement of molecules in the cocrystal lattice can either promote or inhibit chemical degradation processes. For example, the stability of nitrofurantoin under stressful conditions, such as high humidity, elevated temperatures, and UV exposure, was enhanced by co-crystallizing it with 4-hydroxybenzoic acid [50]. The literature typically emphasizes instances where co-crystallization enhances chemical stability; however, it also recognizes that there may be cases where co-crystallization could reduce chemical stability, although such occurrences are seldom documented [51,52].

Mechanical Properties

Co-crystallization can modify crystal packing, leading to improved powder flow, which is essential for many pharmaceutical processes. By altering the crystal habit (shape), co-crystallization enhances flowability [53,54]. Co-crystallization can also improve the compressibility of drug substances, allowing for the creation of tablets with greater hardness and lower friability. For example, paracetamol cocrystals with trimethyl glycine and oxalic acid [55] demonstrated better tableting properties than the pure drug. However, studies highlight that the effect of co-crystallization on mechanical properties is not always consistent and can vary based on the active pharmaceutical moiety, the co-former, and the resulting crystal structure.

Bioavailability

Bioavailability, the portion of a drug or other substance which enters systemic circulation, is a key factor in drug effectiveness. Co-crystallization can greatly improve the bioavailability of poorly soluble drugs by enhancing their solubility and dissolution rate [56,57]. The enhanced dissolution of a drug from a cocrystal can result in higher drug concentrations in the gastrointestinal tract, promoting absorption and boosting bioavailability [58]. The link between co-crystallization and bioavailability is complex. Various factors, such as the cocrystal's dissolution rate, stability in the gastrointestinal environment, and the risk of drug precipitation after dissolution, can affect overall bioavailability. To further improve bioavailability, strategies like maintaining drug supersaturation after cocrystal dissolution - through the use of crystallization inhibitors or amorphous solid dispersions - are being investigated. In summary, it is necessary to emphasize the profound influence of co-crystallization on drug properties and performance, highlighting its potential to address the challenges of poorly soluble drugs and enhance their therapeutic effectiveness. While it presents a promising approach for drug development [59]. Careful selection of the API, co-former, and intended application is essential when using co-crystallization. Ongoing research is focused on understanding the intricate relationship between cocrystal structure and its properties as the field progresses. Below are some examples of cocrystals, along with their respective preparation methods (Table 1).

**Table 1:** Examples of some reported methods of cocrystal preparation

Drug	Co-former	Method of preparation	References
Picolinic acid	Oxalic, succinic, dl-tartaric, pimelic, and phthalic acid	Solvent drop grinding method	[60]
Carbamazepine	Adipic, glutaric, succinic, and malonic acid	Slurry conversion technique	[27]
Rosuvastatin	l-asparagine and l-glutamine	Solvent evaporation technique	[61]
Theophylline anhydrate	Oxalic acid	Spray freeze drying	[62]
Hydrochlorothiazide	Nicotinic acid, nicotinamide, aminobenzoic acid, succinimide, and resorcinol	Liquid-assisted grinding	[63]
Indomethacin	Saccharin	Supercritical fluid technologies	[64]
Modafinil	Sodium acetate, nicotinic acid, benzoic acid, urea, and succinic acetate	Dry grinding method	[65]
Quercetin	Succinic acid	Liquid assisted grinding	[66]
5-fluorouracil	Gentisic acid, 4-aminopyridine	Solvent assisted grinding and Solution crystallization	[67]
Lornoxicam	Puerarin	Solvent evaporation method	[68]
Aceclofenac	Gallic acid, nicotinamide	Solvent evaporation technique	[56]
Acyclovir	Nicotinamide	Solvent evaporation technique	[69]
Lansoprazole	Nicotinamide	Solvent evaporation technique	[70]
Gefitinib	Is nicotinamide, vanillin	Solvent evaporation method	[71]
Zonisamide	Caffeine	Solvent evaporation method	[72]
Nicorandil	Fumaric acid, succinic acid, and oxalic acid	Liquid assisted grinding	[73]
Glibenclamide	Saccharin	Solvent evaporation method	[74]
Mefenamic acid	Ascorbic acid	Solvent evaporation method	[75]
Metaxalone	Lactic acid and saccharin	Solvent evaporation method	[76]
Axitinib	Fumaric acid, suberic acid	Liquid assisted grinding and Slurry method	[77]
Meloxicam	Succinic acid and maleic acid	Solvent drop grinding	[78]
Gliclazide	L-proline, dinitro salicylic acid, and pyridine dicarboxylic acid	Liquid assisted grinding	[79]
Carvedilol	Hydrochlorothiazide	Slurry conversion	[80]

Strategies and methods for cocrystal preparation

This paragraph outlines various design strategies for cocrystals. Cocrystals have gained significant attention in the pharmaceutical and materials science fields owing to their potential for modifying the physical properties of APIs and enhancing their solubility, stability, and bioavailability [81]. The strategies discussed herein include hydrogen bonding propensity, the use of the Cambridge Structural Database (CSD), synthonic engineering, and several other methodologies that facilitate the design and synthesis of cocrystals. The design of cocrystals is a multifaceted process, which benefits from a variety of strategies and methodologies [82]. By utilizing these strategies, researchers can enhance the characteristics of pharmaceutical compounds and develop innovative materials for diverse applications [83].

**Figure 3:**
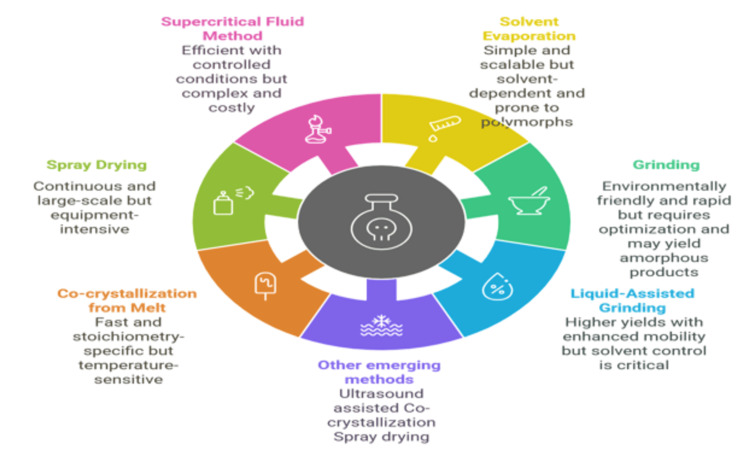
Cocrystal preparation methods (AI-generated) The figure was generated using Napkin AI, an AI-powered tool that converts text-based descriptions into visual representations. This figure categorizes the key techniques for synthesis into three main approaches: (1) solid-based methods, including neat grinding, solvent-assisted grinding, and hot melt extrusion; (2) liquid-based methods, such as solvent evaporation, cooling crystallization, and antisolvent crystallization; (3) other emerging methods, including supercritical fluid technology and spray drying. These methods facilitate the formation of pharmaceutical cocrystals with enhanced physicochemical properties.

Numerous methods for cocrystal preparation have been extensively documented. However, choosing an appropriate co-crystallization technique remains largely empirical. Broadly, the most commonly employed approaches for cocrystal formation (Figure 3) are categorized into solution-based and solid-based methods.

Solid-Based Methods

Among solid-based methods, grinding techniques, including neat grinding (dry grinding) and liquid-assisted grinding (LAG), are widely used and considered more effective than solution or melt techniques [84,85]. Neat grinding involves mixing the API and co-former in a stoichiometric ratio and grinding them, while LAG incorporates a small amount of solvent to enhance molecular diffusion and improve yield. Other solid-state approaches include hot melt extrusion (HME), where the API and co-former are melted, mixed, and extruded into a solid dispersion, making it a solvent-free and continuous process suitable for heat-stable compounds [86,87]. High shear wet granulation, another solid-state method, employs high shear forces and granulating fluid to promote cocrystal formation [88].

Solution-Based Methods

Solution-based methods involve dissolving both the API and co-former in a solvent, followed by various techniques to promote cocrystal formation [89]. Solvent evaporation, a commonly used technique for producing single crystals for X-ray diffraction analysis, involves dissolving the API and co-former in an appropriate solvent or mixture and allowing gradual solvent evaporation, facilitating cocrystal formation. Slow evaporation is preferred to yield larger, high-quality crystals [69,75,90]. Cooling crystallization entails preparing a saturated solution of the active pharmaceutical moiety and co-former at an elevated temperature, followed by gradual cooling, leading to decreased solubility and subsequent crystallization [91,92,93]. Antisolvent crystallization (precipitation) introduces an antisolvent in which the cocrystal has low solubility, reducing its solubility and inducing precipitation. Slurry conversion involves adding the API and co-former in a specific stoichiometric ratio to a solvent and stirring the mixture, maintaining an excess of solid to enable the crystallization of a more thermodynamically stable cocrystal form over time [94]. Reaction crystallization combines individual solutions of the API and co-former in a solvent, leading to their reaction and subsequent cocrystal formation [95]. Supercritical fluid methods, an emerging technology, utilize supercritical fluids like carbon dioxide (CO₂) to enhance cocrystal formation [96]. Co-crystallization with supercritical solvent (CSS) employs supercritical CO₂ as a solvent to suspend the active pharmaceutical moiety and co-former in a slurry, where precise control of pressure and temperature regulates solvent capabilities and co-crystallization [97,98]. The supercritical antisolvent (SAS) technique leverages the antisolvent properties of supercritical CO₂ to induce cocrystal precipitation [64,99,100]. Supercritical fluid-enhanced atomization (SEA) integrates co-crystallization with micronization by dissolving the API and co-former in a supercritical fluid and rapidly expanding the solution through a nozzle, producing fine cocrystal particles with enhanced properties.

Other Emerging Methods

It includes ultrasound-assisted co-crystallization and spray drying. Ultrasound-assisted co-crystallization utilizes ultrasound to enhance the process by promoting nucleation, reducing particle size, and improving yield [101]. Spray drying, a continuous technique, involves spraying a solution of the API and co-former into a heated drying chamber, leading to rapid solvent evaporation and the formation of cocrystal particles. Selecting the appropriate co-crystallization method depends on various factors, including the physical and chemical properties of the API and co-former, the desired cocrystal characteristics such as particle size and morphology, and the feasibility of scaling up for industrial production [26].

Characterization techniques for cocrystals: a detailed overview

**Figure 4:**
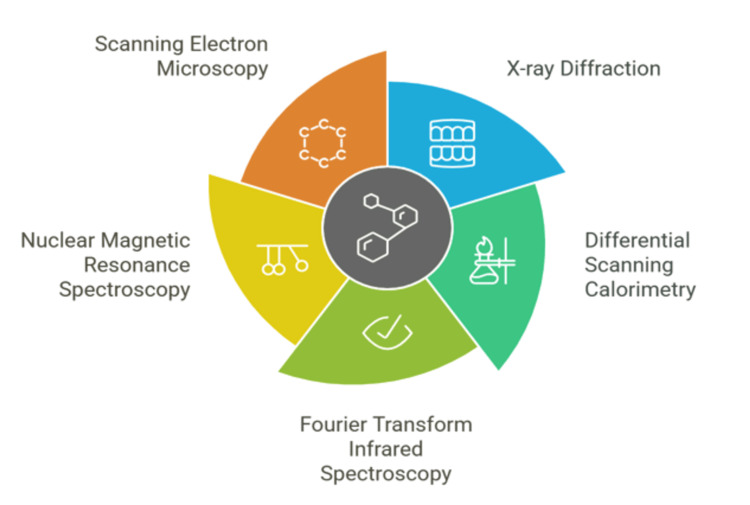
Cocrystal characterization (AI-generated) Schematic representation of cocrystal characterization techniques, including differential scanning calorimetry (DSC), powder X-ray diffraction (PXRD), Fourier-transform infrared spectroscopy (FTIR), nuclear magnetic resonance (NMR), and scanning electron microscopy. The image was generated using Napkin AI, an advanced tool that transforms text-based descriptions into visual illustrations.

Examination of cocrystal characterization highlights the importance of a multifaceted approach to verify their formation and differentiate from other solid forms, such as salts (Figure 4).

Single-Crystal and Powder X-ray Diffraction (XRD)

Techniques like XRD are essential for characterizing cocrystals. Single-crystal XRD, regarded as the gold standard, provides accurate structural information by displaying the arrangement of atoms in the crystal lattice [102]. This technique assists in determining lattice parameters, space groups, Miller indices, unit cell volume, crystal system, and intermolecular and intramolecular interactions.

However, acquiring single crystals of adequate size and quality for analysis is not always possible. In these instances, powder XRD (PXRD) becomes extremely useful. PXRD examines the scattering pattern of X-rays from a powdered sample, yielding information regarding the material's crystallinity. It is especially beneficial for identification since cocrystals display unique, sharp peaks in their diffraction patterns in comparison to their individual components [103].

Assessing the yield of co-crystallization involves calculating the percentage of cocrystals and starting materials present in a sample. Spectroscopic techniques play an important role in characterizing cocrystals and giving valuable information about their intermolecular interactions

FTIR

It is commonly employed to identify and analyze cocrystals because it is fast, non-destructive, and sensitive to changes in molecular structure. It measures the absorption of infrared radiation by the sample, offering insights into the vibrational modes of the molecules [104].

Examining interactions between molecules, especially hydrogen bonds, plays a vital role in the formation of cocrystals [105,106], as well as compatibility studies between the API and co-formers and distinguishing cocrystals from salts. For instance, FTIR can evaluate the involvement of carboxylic acid in hydrogen bonding, which is an essential factor in differentiating cocrystals from salts. THz-TDS is a beneficial method for characterizing cocrystals, complementing PXRD. It analyses the absorption and transmission of terahertz radiation through the sample, yielding a distinctive spectral fingerprint. This technique is especially effective for differentiation purposes like chiral and racemic molecular structures and supramolecular structures.

Solid-state NMR (ss NMR) spectroscopy offers essential insights into the molecular-level properties of cocrystals. It is especially useful when single-crystal XRD analysis is impractical due to challenges in obtaining suitable crystals. In addition, NMR is sensitive to proton transfer, helping distinguish cocrystals from salts and providing a deeper understanding of their structure [107,108].

Thermal Analysis

Thermal analysis involves a group of techniques that examine a substance's behavior in response to temperature changes, offering valuable insights into melting point, phase transitions, and thermal stability.

DSC is a key technique for analyzing cocrystals, enabling researchers to investigate their thermal properties and distinguish them from other solid forms [109]. Important applications of DSC in cocrystal characterization include DSC aids in examining the melting behavior of cocrystals and their constituents. By studying melting point depressions and phase transitions, researchers can identify cocrystal formation and distinguish them from physical mixtures or eutectic mixtures. DSC offers insights into the purity and crystallinity of cocrystals. A sharp endothermic peak with high fusion enthalpy suggests the material is pure and highly crystalline. DSC evaluates the thermal stability of cocrystals, a crucial factor for pharmaceutical applications [110].

Microscopy

HSM is frequently used alongside DSC to visually monitor thermal events in a sample during heating. This method is especially useful for examining melting behavior, crystallization processes, and polymorphic transitions [79,111].

It is essential to recognize that no single technique can comprehensively characterize a cocrystal. A combination of methods is necessary to gain a thorough understanding of its properties and behavior. Experts highlight that employing a multi-technique approach ensures accurate identification, evaluation of purity and stability and supports well-informed decisions in the development of pharmaceutical cocrystals [105].

Future trends in cocrystal research

**Figure 5:**
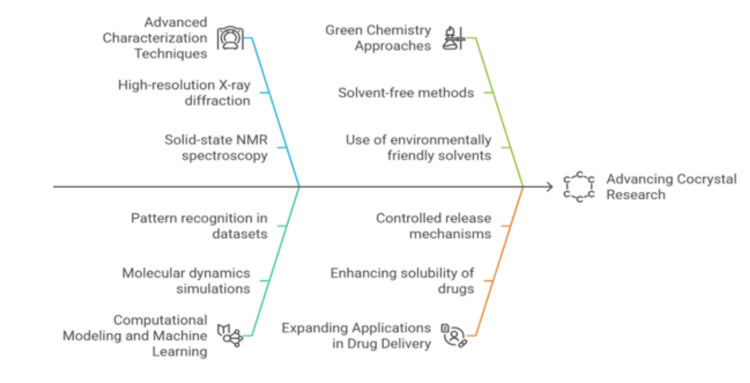
Future trends in cocrystal research (AI-generated) Visualization of future trends in cocrystal research, highlighting advancement in computational screening, high-throughput techniques, regulatory perspectives, green synthesis, solubility enhancement, and controlled release system. The image was created using Napkin AI, an AI-driven tool that converts textual descriptions into visual representations.

Cocrystal research is evolving beyond solubility enhancement, embracing a broader perspective on property modulation, as depicted in Figure 5 [112,113].

Stability studies are increasingly focused on understanding how different co-formers and crystallization conditions affect the physical and chemical stability of cocrystals, including their interactions with excipients and behavior under diverse storage conditions [114]. The mechanical properties of cocrystals remain unpredictable, necessitating future research into optimizing flowability, compressibility, and tableting behavior to improve manufacturability [115]. Taste masking is another growing application, particularly for bitter drugs, where co-crystallization may enhance patient compliance, especially in pediatric formulations [116,117]. Advances in cocrystal design are shifting from empirical screening toward more rational and predictive approaches, with computational cocrystal screening emerging as a vital tool for identifying suitable co-formers and forecasting properties, thereby reducing experimental efforts [118]. The supramolecular synthon approach, which focuses on understanding intermolecular interactions like hydrogen bonding, will be crucial for rational cocrystal design with targeted properties. Beyond binary systems, researchers are increasingly investigating ternary and higher order cocrystals, which offer greater property modulation potential but pose challenges in design, synthesis, and characterization [119]. Salt cocrystals, which blur the line between salts and cocrystals, represent a novel opportunity for ionizable drugs, necessitating further exploration of their synthesis and properties. In addition, polymorphism in cocrystals remains a critical area, as different polymorphs exhibit distinct physicochemical properties, influencing stability and performance [120]. To enable commercial viability, the development of robust and scalable manufacturing processes is imperative, with continuous manufacturing technologies such as hot-melt extrusion, spray drying, and continuous crystallization offering advantages like enhanced process control, reduced waste, and improved product quality. The pursuit of greener, solvent-free co-crystallization methods, including solid-state grinding, supercritical fluid technologies, and ultrasound-assisted crystallization, aligns with sustainability efforts [121]. As cocrystal-based drug products advance toward commercialization, a clear and standardized regulatory framework is essential for characterization, assessment, and approval, alongside addressing intellectual property protection to drive innovation [122]. This dynamic and rapidly evolving field of cocrystal research is driven by the need to overcome the limitations of traditional drug forms, ultimately leading to safer, more effective, and patient-centered medications [122].

## Conclusions

The study collectively highlights the rise of co-crystallization as a significant technique in pharmaceutical science. This method offers a strategic avenue for improving the physicochemical properties of APIs. These improvements mainly focus on solubility, dissolution rate, stability, and mechanical characteristics and pharmacokinetics, which are essential for optimizing drug formulations and enhancing therapeutic efficacy also highlight the importance of co-crystallization in tackling the challenges associated with BCS class II and IV drugs. The review addresses that most of the AEDs fall under BCS class II, where solubility plays a vital role in achieving optimal efficacy. This has led to the development of cocrystals of AEDs, offering an improved solution.

The review explores the mechanisms involved in cocrystal formation and the various synthesis techniques utilized. These techniques include the conventional approach, such as solvent evaporation and reaction crystallization, to more advanced approaches like solid-state grinding, hot-melt extrusion, and supercritical fluid technologies. In addition, it emphasizes the growing significance of rational design strategies, including computational cocrystal screening and the supramolecular synthon approach, in helping select appropriate co-formers and predict cocrystal properties. The review also addresses the changing regulatory landscape concerning pharmaceutical cocrystals. Regulatory agencies have acknowledged the potential of cocrystals and guidelines for their assessment and approval are currently being formulated. Furthermore, it suggests a future in which co-crystallization plays an increasingly vital role in drug development. The advancements in design strategies, manufacturing processes, and a better understanding of cocrystal behavior in biological systems. This progress is expected to aid in the creation of safer, more effective, and patient-focused medications.
